# Poly[tetra­aqua­(μ_6_-9,10-dioxo-9,10-dihydro­anthracene-1,4,5,8-tetra­carboxyl­ato)dimanganese(II)]

**DOI:** 10.1107/S1600536812027158

**Published:** 2012-06-20

**Authors:** Rui Xu, Jian-Lan Liu

**Affiliations:** a Department of Applied Chemistry, College of Science, Nanjing University of Technology, Nanjing 210009, People’s Republic of China

## Abstract

The title complex, [Mn_2_(C_18_H_4_O_10_)(H_2_O)_4_]_*n*_, was synthesized from manganese(II) chloride tetra­hydrate and 9,10-dioxo-9,10-dihydro­anthracene-1,4,5,8-tetra­carb­oxy­lic acid (H_4_AQTC) in water. The anthraquinone unit is located about a crystallographic center of inversion. Each asymmetric unit therefore contains one Mn^II^ atom, two water ligands and one half AQTC^4−^ anion. The Mn^II^ atom is coordinated in a distorted octa­hedral geometry by four O atoms from three AQTC^4−^ ligands and two water O atoms. Two of the carboxyl­ate groups coordinate one Mn^II^ atom in a chelating mode, whereas the others each coordinate two Mn^II^ atoms. Each AQTC^4−^ tetra-anion therefore coordinates six different Mn^II^ ions and, as a result, a three-dimensional coordination polymer is formed. O—H⋯O hydrogen bonds, some of them bifurcated, between water ligands and neighboring water or anthraquinone ligands are observed in the crystal structure.

## Related literature

For general background to metal-organic frameworks, see: Li *et al.* (1999 ▶, 2012 ▶); Cheng *et al.* (2010 ▶); Hong *et al.* (2009 ▶); Miller & Gatteschi (2011 ▶); Liu *et al.* (2010 ▶). 
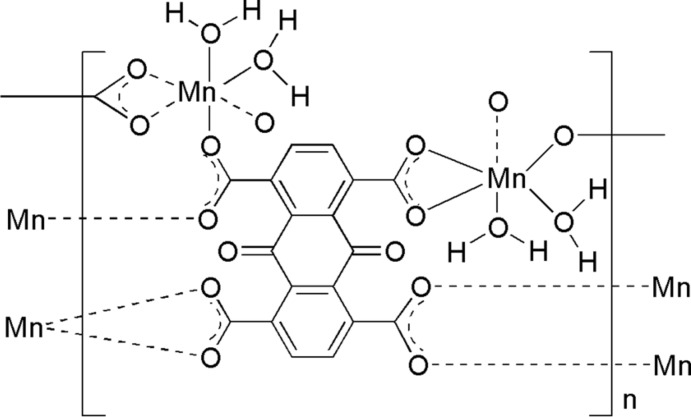


## Experimental

### 

#### Crystal data

[Mn_2_(C_18_H_4_O_10_)(H_2_O)_4_]
*M* *_r_* = 562.16
Monoclinic, 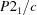

*a* = 11.2255 (16) Å
*b* = 8.4153 (13) Å
*c* = 9.7252 (14) Å
β = 92.355 (2)°
*V* = 917.9 (2) Å^3^
*Z* = 2
Mo *K*α radiation
μ = 1.46 mm^−1^
*T* = 273 K
0.46 × 0.32 × 0.26 mm

#### Data collection

Bruker SMART APEX CCD area-detector diffractometer
Absorption correction: multi-scan (*SADABS*; Bruker, 2000 ▶) *T*_min_ = 0.553, *T*_max_ = 0.702
4340 measured reflections
1609 independent reflections
1499 reflections with *I* > 2σ(*I*)
*R*_int_ = 0.065

#### Refinement

*R*[*F* ^2^ > 2σ(*F* ^2^)] = 0.028
*wR*(*F* ^2^) = 0.076
*S* = 1.08
1609 reflections
170 parameters
2 restraints
H atoms treated by a mixture of independent and constrained refinement
Δρ_max_ = 0.35 e Å^−3^
Δρ_min_ = −0.41 e Å^−3^

### 

Data collection: *SMART* (Bruker, 2000 ▶); cell refinement: *SAINT* (Bruker, 2000 ▶); data reduction: *SAINT*; program(s) used to solve structure: *SHELXS97* (Sheldrick, 2008 ▶); program(s) used to refine structure: *SHELXL97* (Sheldrick, 2008 ▶); molecular graphics: *SHELXTL* (Sheldrick, 2008 ▶); software used to prepare material for publication: *SHELXTL*.

## Supplementary Material

Crystal structure: contains datablock(s) I, global. DOI: 10.1107/S1600536812027158/im2383sup1.cif

Structure factors: contains datablock(s) I. DOI: 10.1107/S1600536812027158/im2383Isup2.hkl

Additional supplementary materials:  crystallographic information; 3D view; checkCIF report

## Figures and Tables

**Table 1:** Hydrogen-bond geometry (Å, °)

*D*—H⋯*A*	*D*—H	H⋯*A*	*D*⋯*A*	*D*—H⋯*A*
O8—H3⋯O2^i^	0.78 (3)	2.03 (3)	2.775 (2)	160 (3)
O8—H4⋯O3^ii^	0.89 (4)	1.86 (4)	2.742 (2)	174 (3)
O8—H4⋯O6^ii^	0.89 (4)	2.60 (3)	3.159 (2)	121 (3)
O9—H5⋯O8^iii^	0.81 (1)	2.03 (1)	2.832 (3)	168 (4)
O9—H6⋯O6^ii^	0.81 (1)	2.38 (2)	3.135 (3)	157 (5)
O9—H6⋯O7^iii^	0.81 (1)	2.50 (4)	3.031 (3)	124 (4)

Symmetry codes: (i) 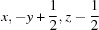; (ii) 
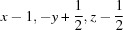
; (iii) 
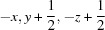
.

**Fig. 1.**
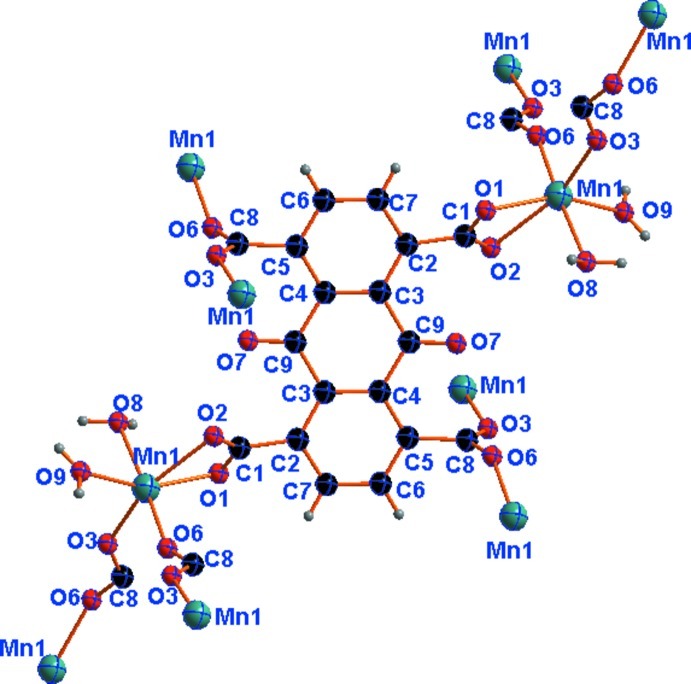
One repeating unit of the coordination polymer, showing displacement ellipsoids at the 30% probability level. [Symmetry codes: (#1) x + 1,-y,-z + 1; (#2) x + 1,y + 1/2,-z + 1/2; (#3) x + 1,-y + 1,-z + 1; (#4) x + 1,y - 1/2,-z + 1/2.]

**Fig. 2.**
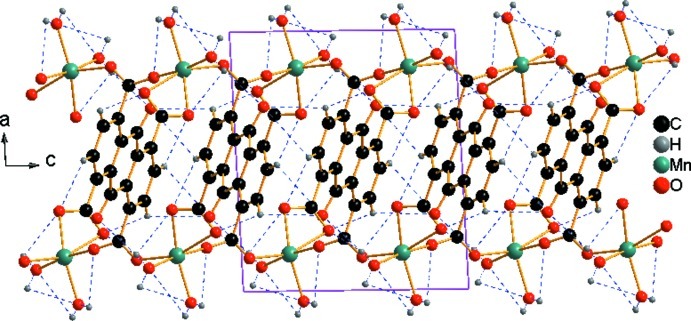
A view of the crystal structure of the title compound.

## supplementary crystallographic information

### Comment

Porous solid materials, such as MOFs (metal-organic frameworks) have been widely studied for their potential applications in gas absorption, separation, catalysis and magnetic materials. Explorations of advanced porous materials for these applications are an intense subject of scientific research (Li *et al.*,1999; Li *et al.*, 2012; Cheng *et al.*, 2010; Hong *et al.*, 2009; Miller & Gatteschi, 2011; Liu *et al.*, 2010.) Herein we report the crystal structure of the title compound.

The molecular structure of (I) is illustrated in Fig. 1., a summary of the observed hydrogen bonds and the corresponding angles are given in Table 1.

Each asymmetric unit therefore contains one manganese(II) atom, two water ligands and one half AQTC^4-^ ligand. The coordination sphere around manganese is distorted octahedral due to the coordination of four O atoms from three AQTC^4-^ ligands and two O atoms from two water molecules. Two of the carboxylate groups coordinate one manganese in a chelating mode whereas the others each coordinate two manganese center. Each AQTC^4- ^therefore coordinates six different manganese ions and as a result a three-dimensional coordination polymer is formed.

### Experimental

A mixture of 9,10-dioxo-9,10-dihydroanthracene-1,4,5,8-tetracarboxylic acid (H_4_AQTC; 0.025 mmol, 9.8 mg) was added to distilled water (4 ml) and ultra-sounded for 10 min. The pH value of the mixture was then adjusted to 7.0 with sodium hydroxide (0.5 mol *L*^-1^), prior to the addition of manganese(II) chloride tetrahydrate (0.05 mmol, 9.9 mg). The reactants were placed in a Teflon-lined stainless steel vessel, heated for 3 days, and then cooled to ambient temperature over 12 h. The solution was exposed to air for three days leading to the precipitation of brown crystals (yield 10%).

### Refinement

All non-hydrogen atoms were refined anisotropically. H atoms of the H_2_O ligands were determined in difference Fourier maps and refined isotropically with distance restraints for O9—H5 and O9—H6 of 0.82 Å. H atoms of AQTC^4-^ ligands calculated in idealized positions with C—H = 0.93 Å and refined as riding atoms, with *U*_iso_(H) = 1.2*U*_eq_(C).

### Crystal data

**Table:**

[Mn_2_(C_18_H_4_O_10_)(H_2_O)_4_]	*F*(000) = 564
*M**_r_* = 562.16	*D*_x_ = 2.034 Mg m^−^^3^
Monoclinic, *P*2_1_/*c*	Mo *K*α radiation, λ = 0.71073 Å
Hall symbol: -P 2ybc	Cell parameters from 5133 reflections
*a* = 11.2255 (16) Å	θ = 2.2–27.6°
*b* = 8.4153 (13) Å	µ = 1.46 mm^−^^1^
*c* = 9.7252 (14) Å	*T* = 273 K
β = 92.355 (2)°	Block, brown
*V* = 917.9 (2) Å^3^	0.46 × 0.32 × 0.26 mm
*Z* = 2	

### Data collection

**Table:**

Bruker SMART APEX CCD area-detector diffractometer	1609 independent reflections
Radiation source: fine-focus sealed tube	1499 reflections with *I* > 2σ(*I*)
Graphite monochromator	*R*_int_ = 0.065
phi and ω scans	θ_max_ = 25.0°, θ_min_ = 3.0°
Absorption correction: multi-scan (*SADABS*; Bruker, 2000)	*h* = −12→13
*T*_min_ = 0.553, *T*_max_ = 0.702	*k* = −10→7
4340 measured reflections	*l* = −11→11

### Refinement

**Table:**

Refinement on *F*^2^	Primary atom site location: structure-invariant direct methods
Least-squares matrix: full	Secondary atom site location: difference Fourier map
*R*[*F*^2^ > 2σ(*F*^2^)] = 0.028	Hydrogen site location: inferred from neighbouring sites
*wR*(*F*^2^) = 0.076	H atoms treated by a mixture of independent and constrained refinement
*S* = 1.08	*w* = 1/[σ^2^(*F*_o_^2^) + (0.0357*P*)^2^ + 0.3487*P*] where *P* = (*F*_o_^2^ + 2*F*_c_^2^)/3
1609 reflections	(Δ/σ)_max_ < 0.001
170 parameters	Δρ_max_ = 0.35 e Å^−^^3^
2 restraints	Δρ_min_ = −0.41 e Å^−^^3^

### Special details

**Table:**

Geometry. All e.s.d.'s (except the e.s.d. in the dihedral angle between two l.s. planes) are estimated using the full covariance matrix. The cell e.s.d.'s are taken into account individually in the estimation of e.s.d.'s in distances, angles and torsion angles; correlations between e.s.d.'s in cell parameters are only used when they are defined by crystal symmetry. An approximate (isotropic) treatment of cell e.s.d.'s is used for estimating e.s.d.'s involving l.s. planes.
Refinement. Refinement of *F*^2^ against ALL reflections. The weighted *R*-factor *wR* and goodness of fit *S* are based on *F*^2^, conventional *R*-factors *R* are based on *F*, with *F* set to zero for negative *F*^2^. The threshold expression of *F*^2^ > σ(*F*^2^) is used only for calculating *R*-factors(gt) *etc*. and is not relevant to the choice of reflections for refinement. *R*-factors based on *F*^2^ are statistically about twice as large as those based on *F*, and *R*- factors based on ALL data will be even larger.

### Fractional atomic coordinates and isotropic or equivalent isotropic displacement parameters (Å^2^)

**Table:**

	*x*	*y*	*z*	*U*_iso_*/*U*_eq_	
C1	0.32095 (18)	0.3515 (2)	0.3203 (2)	0.0199 (4)	
C2	0.44180 (17)	0.2999 (2)	0.37593 (19)	0.0182 (4)	
C3	0.47029 (17)	0.1544 (2)	0.43842 (19)	0.0174 (4)	
C4	0.58569 (16)	0.1290 (2)	0.49497 (19)	0.0168 (4)	
C5	0.67291 (17)	0.2464 (2)	0.4857 (2)	0.0176 (4)	
C6	0.64515 (18)	0.3863 (2)	0.4181 (2)	0.0214 (4)	
H1	0.7036	0.4632	0.4081	0.026*	
C7	0.53093 (18)	0.4128 (2)	0.3654 (2)	0.0214 (4)	
H2	0.5133	0.5087	0.3217	0.026*	
C8	0.79987 (17)	0.2271 (2)	0.5408 (2)	0.0187 (4)	
C9	0.38273 (18)	0.0223 (2)	0.4344 (2)	0.0173 (4)	
Mn1	0.14215 (3)	0.50978 (3)	0.21336 (3)	0.02054 (15)	
O1	0.31881 (13)	0.4118 (2)	0.20209 (15)	0.0305 (4)	
O2	0.23044 (12)	0.35278 (18)	0.39148 (15)	0.0261 (4)	
O3	0.87242 (12)	0.15778 (18)	0.46563 (15)	0.0245 (3)	
O6	0.82943 (13)	0.28802 (18)	0.65405 (15)	0.0274 (4)	
O7	0.28972 (13)	0.03344 (17)	0.36714 (16)	0.0244 (4)	
O8	0.09489 (15)	0.2961 (2)	0.08600 (17)	0.0267 (4)	
O9	−0.04028 (17)	0.5190 (3)	0.2639 (2)	0.0489 (6)	
H3	0.143 (3)	0.273 (4)	0.034 (3)	0.053 (10)*	
H4	0.024 (3)	0.306 (4)	0.042 (4)	0.068 (10)*	
H5	−0.064 (3)	0.590 (3)	0.312 (3)	0.079 (13)*	
H6	−0.091 (3)	0.457 (5)	0.239 (5)	0.114 (17)*	

### Atomic displacement parameters (Å^2^)

**Table:**

	*U*^11^	*U*^22^	*U*^33^	*U*^12^	*U*^13^	*U*^23^
C1	0.0197 (10)	0.0151 (10)	0.0247 (11)	−0.0006 (8)	−0.0031 (8)	0.0011 (8)
C2	0.0176 (10)	0.0206 (10)	0.0166 (10)	0.0002 (8)	0.0007 (7)	0.0003 (8)
C3	0.0157 (9)	0.0201 (10)	0.0163 (10)	0.0020 (8)	0.0004 (7)	−0.0001 (8)
C4	0.0171 (9)	0.0186 (10)	0.0147 (10)	0.0016 (8)	−0.0007 (7)	−0.0010 (7)
C5	0.0172 (10)	0.0191 (10)	0.0163 (10)	0.0015 (8)	−0.0002 (7)	−0.0031 (8)
C6	0.0183 (10)	0.0225 (11)	0.0233 (11)	−0.0042 (8)	0.0000 (8)	0.0011 (8)
C7	0.0223 (10)	0.0191 (10)	0.0225 (10)	0.0012 (8)	−0.0008 (8)	0.0042 (8)
C8	0.0177 (10)	0.0154 (10)	0.0228 (11)	−0.0029 (8)	−0.0027 (8)	0.0029 (8)
C9	0.0158 (10)	0.0183 (10)	0.0178 (10)	0.0016 (8)	0.0002 (8)	−0.0015 (8)
Mn1	0.0173 (2)	0.0225 (2)	0.0215 (2)	0.00180 (12)	−0.00320 (14)	−0.00052 (12)
O1	0.0221 (8)	0.0426 (10)	0.0265 (8)	0.0031 (7)	−0.0023 (6)	0.0113 (7)
O2	0.0205 (8)	0.0280 (8)	0.0299 (8)	0.0030 (6)	0.0035 (6)	0.0038 (6)
O3	0.0166 (7)	0.0319 (8)	0.0247 (8)	0.0000 (6)	−0.0016 (6)	−0.0063 (6)
O6	0.0260 (8)	0.0289 (8)	0.0266 (8)	−0.0003 (7)	−0.0069 (6)	−0.0097 (7)
O7	0.0183 (8)	0.0225 (7)	0.0315 (9)	0.0000 (6)	−0.0105 (6)	0.0041 (6)
O8	0.0185 (8)	0.0339 (9)	0.0276 (9)	0.0005 (7)	−0.0007 (7)	−0.0065 (7)
O9	0.0217 (9)	0.0638 (14)	0.0617 (14)	−0.0095 (9)	0.0059 (9)	−0.0353 (11)

### Geometric parameters (Å, º)

**Table:**

C1—O2	1.253 (3)	C8—O3	1.260 (2)
C1—O1	1.256 (2)	C9—O7	1.212 (2)
C1—C2	1.504 (3)	C9—C4^i^	1.483 (3)
C2—C7	1.386 (3)	Mn1—O9	2.1270 (19)
C2—C3	1.398 (3)	Mn1—O3^ii^	2.1412 (15)
C3—C4	1.403 (3)	Mn1—O6^iii^	2.1508 (15)
C3—C9	1.483 (3)	Mn1—O1	2.1547 (15)
C4—C5	1.396 (3)	Mn1—O8	2.2350 (16)
C4—C9^i^	1.483 (3)	Mn1—O2	2.3637 (15)
C5—C6	1.378 (3)	O3—Mn1^iv^	2.1412 (15)
C5—C8	1.511 (3)	O6—Mn1^iii^	2.1508 (15)
C6—C7	1.379 (3)	O8—H3	0.78 (3)
C6—H1	0.9300	O8—H4	0.89 (4)
C7—H2	0.9300	O9—H5	0.812 (10)
C8—O6	1.247 (2)	O9—H6	0.809 (10)
			
O2—C1—O1	121.11 (18)	C4^i^—C9—C3	119.05 (17)
O2—C1—C2	122.96 (18)	O9—Mn1—O3^ii^	97.16 (8)
O1—C1—C2	115.40 (17)	O9—Mn1—O6^iii^	87.31 (7)
C7—C2—C3	118.63 (18)	O3^ii^—Mn1—O6^iii^	91.81 (6)
C7—C2—C1	114.76 (17)	O9—Mn1—O1	157.30 (8)
C3—C2—C1	126.59 (18)	O3^ii^—Mn1—O1	102.77 (5)
C2—C3—C4	119.69 (18)	O6^iii^—Mn1—O1	102.67 (6)
C2—C3—C9	120.34 (17)	O9—Mn1—O8	87.05 (7)
C4—C3—C9	119.75 (18)	O3^ii^—Mn1—O8	90.52 (6)
C5—C4—C3	120.36 (18)	O6^iii^—Mn1—O8	174.13 (6)
C5—C4—C9^i^	118.84 (17)	O1—Mn1—O8	82.05 (6)
C3—C4—C9^i^	120.80 (17)	O9—Mn1—O2	103.28 (8)
C6—C5—C4	119.34 (17)	O3^ii^—Mn1—O2	159.49 (5)
C6—C5—C8	116.90 (17)	O6^iii^—Mn1—O2	87.45 (6)
C4—C5—C8	123.71 (17)	O1—Mn1—O2	57.61 (5)
C5—C6—C7	120.24 (19)	O8—Mn1—O2	92.25 (6)
C5—C6—H1	119.9	C1—O1—Mn1	95.23 (12)
C7—C6—H1	119.9	C1—O2—Mn1	85.71 (12)
C6—C7—C2	121.64 (19)	C8—O3—Mn1^iv^	135.36 (13)
C6—C7—H2	119.2	C8—O6—Mn1^iii^	151.88 (14)
C2—C7—H2	119.2	Mn1—O8—H3	114 (2)
O6—C8—O3	123.22 (18)	Mn1—O8—H4	112 (2)
O6—C8—C5	118.82 (17)	H3—O8—H4	110 (3)
O3—C8—C5	117.82 (17)	Mn1—O9—H5	120 (3)
O7—C9—C4^i^	120.01 (18)	Mn1—O9—H6	126 (4)
O7—C9—C3	120.77 (18)	H5—O9—H6	114 (5)
			
O2—C1—C2—C7	−122.6 (2)	C4—C5—C8—O3	84.0 (2)
O1—C1—C2—C7	49.1 (3)	C2—C3—C9—O7	6.4 (3)
O2—C1—C2—C3	55.6 (3)	C4—C3—C9—O7	−168.17 (19)
O1—C1—C2—C3	−132.7 (2)	C2—C3—C9—C4^i^	−178.27 (17)
C7—C2—C3—C4	3.3 (3)	C4—C3—C9—C4^i^	7.1 (3)
C1—C2—C3—C4	−174.94 (18)	O2—C1—O1—Mn1	6.2 (2)
C7—C2—C3—C9	−171.33 (17)	C2—C1—O1—Mn1	−165.70 (15)
C1—C2—C3—C9	10.5 (3)	O9—Mn1—O1—C1	−39.0 (2)
C2—C3—C4—C5	−1.8 (3)	O3^ii^—Mn1—O1—C1	170.17 (12)
C9—C3—C4—C5	172.81 (17)	O6^iii^—Mn1—O1—C1	75.34 (13)
C2—C3—C4—C9^i^	178.11 (17)	O8—Mn1—O1—C1	−101.11 (13)
C9—C3—C4—C9^i^	−7.3 (3)	O2—Mn1—O1—C1	−3.32 (11)
C3—C4—C5—C6	−1.2 (3)	O1—C1—O2—Mn1	−5.63 (19)
C9^i^—C4—C5—C6	178.86 (18)	C2—C1—O2—Mn1	165.63 (18)
C3—C4—C5—C8	−178.52 (18)	O9—Mn1—O2—C1	169.94 (12)
C9^i^—C4—C5—C8	1.6 (3)	O3^ii^—Mn1—O2—C1	−15.1 (2)
C4—C5—C6—C7	2.8 (3)	O6^iii^—Mn1—O2—C1	−103.43 (12)
C8—C5—C6—C7	−179.76 (18)	O1—Mn1—O2—C1	3.32 (11)
C5—C6—C7—C2	−1.3 (3)	O8—Mn1—O2—C1	82.44 (12)
C3—C2—C7—C6	−1.8 (3)	O6—C8—O3—Mn1^iv^	168.36 (14)
C1—C2—C7—C6	176.64 (19)	C5—C8—O3—Mn1^iv^	−15.9 (3)
C6—C5—C8—O6	82.5 (2)	O3—C8—O6—Mn1^iii^	112.0 (3)
C4—C5—C8—O6	−100.1 (2)	C5—C8—O6—Mn1^iii^	−63.7 (4)
C6—C5—C8—O3	−93.4 (2)		

Symmetry codes: (i) −*x*+1, −*y*, −*z*+1; (ii) −*x*+1, *y*+1/2, −*z*+1/2; (iii) −*x*+1, −*y*+1, −*z*+1; (iv) −*x*+1, *y*−1/2, −*z*+1/2.

### Hydrogen-bond geometry (Å, º)

**Table:**

*D*—H···*A*	*D*—H	H···*A*	*D*···*A*	*D*—H···*A*
O8—H3···O2^v^	0.78 (3)	2.03 (3)	2.775 (2)	160 (3)
O8—H4···O3^vi^	0.89 (4)	1.86 (4)	2.742 (2)	174 (3)
O8—H4···O6^vi^	0.89 (4)	2.60 (3)	3.159 (2)	121 (3)
O9—H5···O8^vii^	0.81 (1)	2.03 (1)	2.832 (3)	168 (4)
O9—H6···O6^vi^	0.81 (1)	2.38 (2)	3.135 (3)	157 (5)
O9—H6···O7^vii^	0.81 (1)	2.50 (4)	3.031 (3)	124 (4)

Symmetry codes: (v) *x*, −*y*+1/2, *z*−1/2; (vi) *x*−1, −*y*+1/2, *z*−1/2; (vii) −*x*, *y*+1/2, −*z*+1/2.
